# Resection of dominant fusiform gyrus is associated with decline of naming function when temporal lobe epilepsy manifests after the age of five: A voxel-based lesion-symptom mapping study^☆^

**DOI:** 10.1016/j.nicl.2022.103129

**Published:** 2022-07-29

**Authors:** Caroline Reindl, Anna-Lena Allgäuer, Benedict A. Kleiser, Müjgan Dogan Onugoren, Johannes D. Lang, Tamara M. Welte, Jenny Stritzelberger, Klemens Winder, Michael Schwarz, Stephanie Gollwitzer, Regina Trollmann, Julie Rösch, Arnd Doerfler, Karl Rössler, Sebastian Brandner, Dominik Madžar, Frank Seifert, Stefan Rampp, Hajo M. Hamer, Katrin Walther

**Affiliations:** a Department of Neurology, University Hospital Erlangen, Erlangen, Germany; b Department of Neurosurgery, University Hospital Erlangen, Erlangen, Germany; c Department of Neuroradiology, University Hospital Erlangen, Erlangen, Germany; d Department of Neuropaediatrics, University Hospital Erlangen, Erlangen, Germany; e Department of Neurology, Hertie-Institute for Clinical Brain Research, University of Tübingen, Tübingen, Germany; f Department of Neurology, University Hospital Zürich (USZ), Zürich, Switzerland; g Department of Neurosurgery, University Hospital Vienna (AKH), Vienna, Austria; h Department of Neurosurgery, University Hospital Halle (Saale), Germany

**Keywords:** AH, amygdalohippocampectomy, ATL, anterior temporal lobectomy, BNT, Boston Naming Test, BTLA, basal temporal language area, CI, confidence interval, EEG, electroencephalography, fMRI, functional magnetic resonance imaging, FG, fusiform gyrus, ITG, inferior temporal gyrus, IQR, Interquartile range, OR, Odds ratio, SD, standard deviation, VBLSM, voxel-based lesion-symptom mapping, Epilepsy surgery, Lesion localization, Basal temporal language area, Gyrus fusiformis, Naming, Neuropsychological outcome

## Abstract

**Objective:**

To determine patients’ characteristics and regions in the temporal lobe where resections lead to a decline in picture naming.

**Methods:**

311 patients with left hemispheric dominance for language were included who underwent epilepsy surgery at the Epilepsy Center of Erlangen and whose picture naming scores (Boston Naming Test, BNT) were available preoperatively and 6-months postoperatively. Surgical lesions were mapped to an averaged template based on preoperative and postoperative MRI using voxel-based lesion-symptom mapping (VBLSM). Postoperative brain shifts were corrected. The relationship between lesioned brain areas and the presence of a postoperative naming decline was examined voxel-wise while controlling for effects of overall lesion size at first in the total cohort and then restricted to temporal lobe resections.

**Results:**

In VBLSM in the total sample, a decline in BNT score was significantly related to left temporal surgery. When only considering patients with left temporal lobe resections (n = 121), 40 (33.1%) significantly worsened in BNT postoperatively. VBLSM including all patients with left temporal resections generated no significant results within the temporal lobe. However, naming decline of patients with epilepsy onset after 5 years of age was significantly associated with resections in the left inferior temporal (extent of BNT decline range: 10.8− 14.4%) and fusiform gyrus (decline range: 12.1−18.4%).

**Significance:**

Resections in the posterior part of the dominant fusiform and inferior temporal gyrus was associated with a risk of deterioration in naming performance at six months after surgery in patients with epilepsy onset after 5 years of age but not with earlier epilepsy onset.

## Highlights

• Resection in the dominant fusiform gyrus is associated with an increased risk of postoperative decline in picture naming.
• More temporo-posterior resections in this area results in a greater degree of naming decline.
• Risk of significant naming decline after left temporal surgery increased by 5% with every year of later seizure onset.

## 1 Introduction

Epilepsy surgery is an effective procedure for temporal lobe epilepsies refractory to medication with a number needed to treat of two (Wiebe et al., 2001, Josephson et al., 2013). Resection strategies increasingly allow a tailored approach selective to the epileptogenic zone (Drane et al., 2015, Ross et al., 2018, Liu et al., 2021). The extent of resection is a tradeoff of sufficient removal of the epileptogenic zone and prevention of cognitive deficits (Helmstaedter, 2013, Baxendale and Thompson, 2018). A recent study showed that epilepsy surgery was not pursued due to expected postoperative deficits in 11% of epilepsy surgical evaluations (Khoo et al., 2021). Postoperative decrease in language functions such as naming is one of the neuropsychological deficits which clinicians are worried about when counselling surgical candidates (Krauss et al., 1996). On average, a naming decline was found in one third of patients after left-sided temporal surgery (Sherman et al., 2011, Helmstaedter, 2013). However, it is still not entirely clear where specific language functions are located in the left temporal lobe and in particular how critical a damage is to be able to advise patients well and to plan tailored resections optimally.

Lüders et al. identified a basal temporal language area (BTLA) as a language processing area, mainly responsible for confrontational naming (Lüders et al., 1991). The BTLA was delineated by high-frequency cortical stimulation during stereo-EEG 10 to 90 mm from the temporal pole, primarily located in the fusiform gyrus, extending into the parahippocampal gyrus, the inferior temporal gyrus, and the occipito-temporal sulcus (Krauss et al., 1996, Bédos Ulvin et al., 2017, Abdallah et al., 2021). Studies applying fMRI showed functional connectivity of the area around the gyrus fusiformis with attentional, visual and auditoy-sensory, lexical semantic processing, and articulation, reflecting the involvement in a language network (Binder et al., 2009, Forseth et al., 2018, Chen et al., 2019). In patients with left temporal resections, factors predicting a postoperative impaired function of language were an onset of epilepsy after early childhood, in particular after the age of 5, a typical organization of language, older age at surgery, and high baseline naming performance (Krauss et al., 1996, Helmstaedter and Witt, 2012, Busch et al., 2016).

Voxel-based lesion-symptom mapping (VBLSM) has shown to be a powerful tool to identify brain regions necessary for specific cognitive processes (Rorden et al., 2007). A recent lesion-symptom mapping study found the fusiform gyrus as an essential language area for visual naming (Binder et al., 2020). The results were obtained from a study cohort of 65 epilepsy patients that underwent dorsally extending left temporal resections and often had a postoperative naming deficit.

In our study, we examined postoperative naming decline in patients who underwent epilepsy surgery using voxel-based lesion-mapping It has been shown that deterioration of naming can also occur in regions outside of the left temporal lobe but not to the same extent (Busch et al., 2016). To test for a systematic association between lesion localisation and naming decline also outside the left temporal region, we first performed VBLSM in all patients without restrictions to the lesion location. In a second step, we focussed on left temporal patients. We aimed not only to identify the brain region essential for naming function in a large cohort of patients but also to investigate the influence of patient characteristics, such as age at epilepsy onset, on the localization of naming function and to quantify the extent of postoperative naming impairment in relation to the extent of resection.

## 2 Methods

### 2.1 Patients

We identified all patients who underwent epilepsy surgery at the Epilepsy Center of Erlangen between 1998 and 2020. As part of their presurgical assessment, all patients underwent video-EEG monitoring, 3 T or 1.5 T MRI, and neuropsychological testing. In addition, postoperative follow-up included repeat MRI and a neuropsychological re-testing at 6 months after surgery. Inclusion criteria comprised availability of a pre- and postoperative 3D data set of T1 weighted MRI as well as Boston naming test (BNT) (Kaplan et al., xxxx) and left hemispheric dominance for language. Lateralization of language function was based on neuropsychological testing, handedness, and supplementary WADA testing and fMRI of speech, if available. Postoperative seizure outcomes were scored according to Engel classification (Engel, 1992). All patients gave written general informed consent for participating in scientific studies. Our institutional ethical review committee approved the conduct of this non-interventional study in a retrospective design and waived an ethics application.

### 2.2 Pre- and postsurgical neuropsychological assessment

The Boston naming test, a tool to examine picture naming ability, is part of the standard pre- and postoperative neuropsychological evaluation (Baxendale et al., 2019, Kaplan et al.). The assessments were performed and supervised by trained staff neuropsychologists. Patients were requested to name one by one a series of 60 line-drawings of increasing difficulty. An item was named correctly when the patient’s response was given within a window of 5–7 s after presentation and did not deviate from the target name either semantically or phonologically. The test was discontinued after half of the drawings if patients had six or more consecutive errors at easy and medium difficulty due to severe anomia. In our cohort, this was the case in five patients with left temporal epilepsy and three patients with right temporal epilepsy. Overall test performance was calculated as the sum score of correctly named items. A pre- and postoperative score of 48 was considered as unimpaired (Nicholas et al., 1988).

A significant clinically meaningful change in BNT was defined by using an epilepsy specific reliable change indices (RCI) of ≥ 5 offered by Sawrie et al. (1996) (Sawrie et al., 1996). One drawback of a difference score is that it does not consider the baseline level and therefore, underestimates the level of impairment in particular in patients with low baseline performance. We additionally calculated a percent change score by taking into account the preoperative score (100*(correct items postoperative – correct items preoperative) / correct items preoperative) and considered similar to Binder 10% as moderate decline (Binder et al., 2020). This change score of 10% was approximately equivalent to the RCI of ≥ 5.

### 2.3 Resection types

All mesial temporal resections encompassed the amygdala and most of the hippocampus and were classified as standard anterior temporal lobectomy (ATL), a more tailored resection of the temporal pole, and an amygdalohippocampectomy (pole + AH) when removal of anterior/posterior neocortex was more limited, and selective amygdalohippocampectomy (selective AH) (Zentner, 2016, Wiebe et al., 2001, Josephson et al., 2013). The extent of the lateral neocortical resection varied in our cohort.

### 2.4 Imaging and lesion delineation

For all patients in this study, magnetic resonance imaging was performed according to a standard epilepsy protocol of the Epilepsy Center of Erlangen (3 Tesla, Magnetom Trio or in 15% of cases 1.5 Tesla Magnetom Sonata, Siemens Healthcare, Erlangen, Germany, ∼9% changed from 1.5 T (pre-) to 3 T (postsurgery). High resolution T1-weighted 3D datasets with a resolution of 1 × 1 × 1 mm were acquired utilizing a MP-RAGE sequence. Lesion mapping was carried out using SPM 12 (https://www.fil.ion.ucl.ac.uk/spm) and following the method of Rorden (2012) and Karnath (2019) (Rorden et al., 2012, Karnath et al., 2020). First, pre- and postoperative T1 sequences were aligned in reference to the anterior and posterior commissure and co-registered to each other. T1 images were then resliced to a 2 × 2 × 2 mm resolution for manual tracing of the lesion. Lesions were delineated slice by slice on the postoperative T1 image using MRIcron software (https://www.mricro.com) (Rorden and Brett, xxxx) starting in the coronal plane and editing in axial and sagittal view. Displacement of brain tissue into the resection cavity is a common problem in particular with increasing resection volume. To correct for these postoperative shifts the lesion mask was overlaid on the preoperative T1. The lesion mask was then extended or adjusted based on the preoperative outline of the gyri, sulci, midline, ventricle, or mesial structures of the hippocampus and amygdala. Postoperative T1 and lesion masks were then transformed into standard stereotactic space (MNI) using the ‘clinical toolbox’ implemented in SPM12 (https://www.nitrc.org/projects/clinicaltbx/). Images were normalized to a template of an older adult using enantiomorphic lesion masking to minimize normalisation artefacts by replacing the lesion with brain tissue from the intact contralateral hemisphere and unified normalization-segmentation routines (Nachev et al., 2008, Ashburner and Friston, 2005). The normalized T1 scans and lesion map were resampled to 1 × 1 × 1 mm voxels, visually inspected for quality and in case of failed normalisation excluded from further analysis. When overlaying lesion maps of all patients we noticed in some patients that the lesions did not expand to the border of the skull (e.g. basal region of the temporal lobe). To correct for this mismatch, an average T1 image of 200 patients with high quality normalized T1 was created. This template was used to generate a binary mask to define common outer borders of the brain. Lesion maps were overlapped with this mask and missing lesion volumes to the borders was manually added.

The resulting normalized lesion maps were used for statistical analyses.

### 2.5 Statistical analysis and voxel-based lesion-symptom mapping

Lesion-symptom mapping was calculated with NiiStat (https://www.nitrc.org/projects/niistat). VBLSM analyses to calculate correlation of lesion site and occurrence of naming deficits (yes/no) were corrected for the lesion volume and restricted to voxel resected in at least 5 patients. Analyses used the Freedman-Lane test and 10,000 permutations to correct for multiple comparisons and thresholds were one tailed (p <0.05, with an extend threshold of 50 contiguous voxels).

Coordinates of significant clusters were presented in the Montreal Neurological Institute (MNI) space and displayed on the MNI152 standard-space T1-weighted average structural template image for 3D-visualization in MRIcroGL (https://www.nitrc.org/projects/mricrogl) (Rorden and Brett, xxxx).

We calculated the extent of naming decline within the significant cluster, using a MatLab script. For this purpose, the extent of naming decline was transferred to the volume of interest as a point value. The mean value of all significant voxels of interest corresponding to the VBLSM was then color-coded for visualization.

Statistics of demographic and behavioral data were performed using IBM SPSS Statistics 22.0 (http://www.spss.com). Statistical analysis of differences between groups were only performed in patients with left temporal epilepsies. First, deterioration of BNT (yes, no) was used as group factor to identify potential factors for patients on risk of BNT deterioration. Proportions were compared using Pearson’s Chi2 test, continuous variables were compared using the Mann-Whitney *U* test. Variables showing significant group differences were considered candidate covariates for multivariate analyses using binary logistic regression to identify independent predictors. Further, we examined the effect of seizure onset and computed group comparisons between patients with onset before and after 5 years of age. Two-sided p values of <0.05 were considered statistically significant. We used the STROBE cohort checklist when writing our report (von Elm et al., 2008).

## 3 Results

### 3.1 Total patient group

**Fig. 1:**
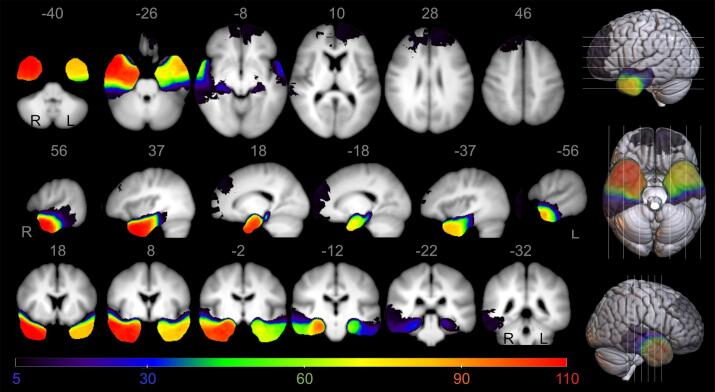
Lesion overlap map of total patient group showing regions with lesions in at least 5 patients. Coordinates are presented in MNI space, results are presented on an averaged T1 brain template of our cohort in axial, sagittal and coronal view, 3D-visualization is presented on a standard MNI152 template. Color bar visualizes number of patients with overlapping resection zones. R: right; L: left.

**Fig. 2:**
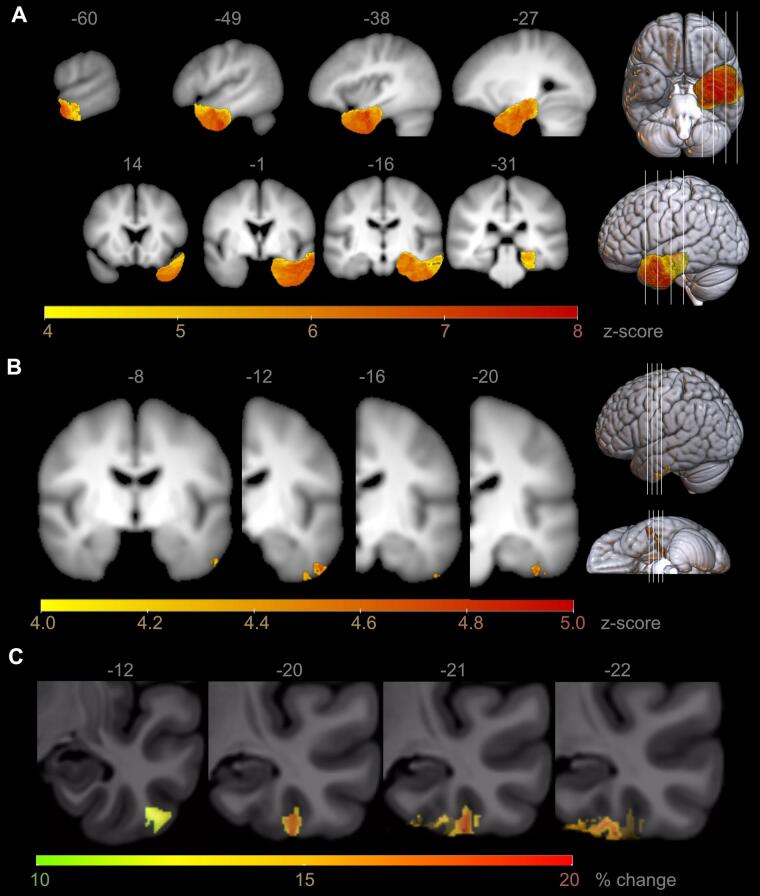
Voxel-based lesion-symptom mapping (VBLSM) showing regions associated with significant postoperative deterioration in picture naming. Coordinates are presented in MNI space, results are presented on an averaged T1 brain template of our cohort in a sagittal and coronal view, 3D-visualization and magnified view of the left temporal lobe are presented on a standard MNI152 template. **A)** All patients, p <0.01. Voxels with significant correlation are colour coded according to their z-value. **B)** Only patients with left temporal epilepsy surgery and onset of epilepsy ≥5 years, p <0.05. Voxels with significant correlation are colour coded according to the z-value. **C)** Extent of naming decline in percent in regions with significant correlation of surgical resection and postoperative picture naming performance, magnified to left temporal lobe in coronal view.

A total of 311 epilepsy patients could be included (female: 162 (52.1%), age: median 37, IQR 27 – 49 or mean 38 +/-12.8 SD). Of our total cohort, 278 patients (89%) were right handed and Wada/fMRI was performed in 105 patients (34%). Not surprisingly, Wada/fMRI were most often carried out in patients with left temporal epilepsies (53%) or left handed/ ambidextrous patient (70%; for a more detailed breakdown see Supplementary information, Table 2). One hundred and twenty-one patients had left temporal lobe resections, 20 patients left extra-temporal resections (15 frontal, 4 parietal, 1 occipital), 140 patients right temporal resections, and 30 patients right extra-temporal resections (21 frontal, 6 parietal, 3 occipital) (see Table 1). Fig. 1 shows the lesion overlap for all patients thresholded to show only voxels resected in at least 5 patients. Fig. 2A illustrates the result of VBLSM analysis, showing that postoperative deterioration in naming performance in the total patient group was significantly associated with left temporal resection in 86,833 voxels (p <0.05).

**Table 1:** Patients’ characteristics.

	Left temporal resection	Left extratemporal resection	Right temporal resection	Right extratemporal resection
N	121	20	140	30
Female gender, n(%)	60 (49.6)	6 (30.0)	79 (56.4)	17 (56.7)
Age at epilepsy onset, m(range)	21.0 (0–62)	16.5 (3–53)	17.0 (0–59)	16.5 (2–52)
Age at surgery, m(range)	38.0 (17–68)	30.0 (20–54)	39.0 (18–67)	29.0 (18–53)
Years between epilepsy onset and surgery, m(range)	13.0 (0–57)	13.5 (0–37)	18.0 (0–59)	11.0 (1–35)
Normalized lesion volume in ml, m(range)	27.8 (0.3–63.3)	29.8 (1.4–110.5)	44.2 (0.3–100.5)	12.6 (0.6–112.9)
BNT decline greater than 10 %, n(%)	40 (33.1)	3 (15.0)	3 (2.1)	1 (3.3)
6 months Outcome Engel class 1, n(%)	96 (79.3)	12 (63.2)	105 (75.0)	24 (80.0)

Abbreviations: n number, m median, BNT Boston Naming Test.

### 3.2 Patients with left temporal resection

Patients with left temporal lobe resections worsened by a mean of 6.0% (15.0% SD) in BNT 6 months postoperatively. The degree of deterioration was widely distributed within the group; 33.1% (n = 40) deteriorated by more than 10%, 14.9% (n = 18) showed deterioration by more than 20%, and 5.8% (n = 7) showed deterioration by more than 30%. In univariate analyses (Table 2), patients with postoperative deterioration of BNT were significantly older at the time of epilepsy manifestation as well as at the time of surgery, and received more extensive resections and more often a mesial temporal resection. Histopathology was not significantly associated with postoperative naming deficit. The postoperative seizure outcome was not significantly different. Due to the high intercorrelation between lesion volume and resection type, we only included lesion volume as potential predictor. In multivariate analysis, only age at seizure onset and lesion size survived as independent predictors for postoperative BNT decline (goodness of fit Nagelkerke 0.28) (Table 2). The risk of postoperative decline of naming increased by 5% with each year of later initial manifestation and also with each ml of resected volume.

**Table 2:** Postoperative decline in picture naming in patients with left temporal resections and logistic regression for BNT.

	No decline of BNT (n = 81)	BNT decline (n = 40)	Uni-variate analysis p value	Logistic regression OR (95% CI)	Logistic regression p value
Demographics					
Female gender, n(%)	42 (51.9)	18 (45.0)	0.480		
Years of education, m(IQR)	10 (8 – 12)	10 (8 – 10)	0.201		
Age at surgery, m(IQR)	36 (24 – 44)	48 (34 – 55)	**0.0006**	1.03 (0.990 – 1.069)	0.148
Epilepsy					
Age at onset, m(IQR)	16 (9 – 26)	28 (20 – 39)	**0.0005**	1.05 (1.011 – 1.087)	**0.011**
Years between epilepsy onset and surgery, m(IQR)	12 (4–26)	15 (5 – 27)	0.589		
Histopathology					
Hippocampus sclerosis, n(%)	25 (31.2)	15 (36.6)	0.557		
Tumor, n(%)	39 (48.7)	15 (36.6)	0.205		
Non-lesional, n(%)	10 (12.5)	11 (26.8)	0.05		
Other pathology, n(%)	6 (7.5)	2 (4.9)	0.584		
BNT preoperative normal, n(%)	49 (60.5)	23 (57.5)	0.753		
Normalized lesion volume in ml, m(IQR)	24.4 (7.5 – 36.5)	34.5 (22.5 – 43.0)	**0.002**	1.05 (1.014 – 1.080)	**0.004**
6 months Outcome Engel class 1, n(%)	68 (84.0)	28 (70.0)	0.076		

Statistically significant values (p <0.05) are expressed in bold. Abbreviations: n number, m median, IQR interquartile range, BNT Boston Naming Test, OR Odds ratio, CI confidence interval.

Surgical resection types varied in our cohort of temporal lobe epilepsy. Table 1 in the supplementary material specifies neuropsychological outcome by resection type.

Considering only patients with left temporal lobe resections, we could not identify significant voxels within the temporal lobe across subjects in VBLSM analysis which predicted decline in naming performance. We hypothesized that this finding may reflect individual language allocation possibly influenced by the individual expression of the epilepsy in the plastic first years of life.

### 3.3 Patients with epilepsy onset before and after 5 years of age

We repeated lesion mapping including only patients with epilepsy onset ≥ 5 years of age. This was done to minimize effects of possible language reorganization due to early manifestation of the epilepsy (Krauss et al., 1996, Berl et al., 2014). Thus, we excluded 13 patients. These excluded patients showed a significantly lower level of education in school years and were significantly more likely to have a poor preoperative naming performance (Table 3). Patients with onset of epilepsy before 5 years of age had significantly more extensive resections in terms of lesion volume. In addition, the duration of epilepsy at the time of surgery was significantly longer when epilepsy began in early childhood.

**Table 3:** Demographics of patients with left temporal resections and early versus later epilepsy onset.

	**Age at onset < 5** **(n = 13)**	**Age at onset ≥ 5** **(n = 108)**	**p value**
Demographics			
Female gender, n(%)	5 (38.5)	57 (50.9)	0.398
Years of education, m(IQR)	8 (8 – 9)	10 (8 – 12)	**0.0006**
Age at surgery, m(IQR)	34 (25 – 40)	39 (28 – 50)	0.243
Epileptic syndrome			
Years between epilepsy onset and surgery, m(IQR)	32 (23 – 40)	11 (4 – 22)	**<0.0001**
Age at onset, m(IQR)	2 (1 – 3)	23 (15 – 36)	**<0.0001**
Neuropsychological results			
BNT preoperative normal, n(%)	4 (30.8)	68 (63.0)	**0.026**
BNT postoperative decline, n(%)	2 (15.4)	38 (35.2)	0.153
Resection type			
Temporal neocortical, n(%)	5 (38.5)	51 (47.2)	0.551
Temporal mesial, n(%)	8 (61.5)	57 (52.8)	0.551
Normalized lesion volume in ml, m(IQR)	36.6 (24.8 – 46.6)	26.6 (13.3 – 37.1)	**0.031**
6 months Outcome Engel class 1, n(%)	9 (69.2)	87 (80.6)	0.343

Statistically significant values (p <0.05) are expressed in bold. Abbreviations: n number, m median, IQR interquartile range, BNT Boston Naming Test.

VBLSM of patients with onset of epilepsy after 5 years of age showed a significant correlation of BNT decline and lesions in the left temporo-basal area for 587 voxels (p <0.05; Fig. 2B). In standardized stereotaxic space, the cluster was located in the inferior temporal gyrus (ITG) extending from 34 mm from the temporal pole to 40 mm posterior of the pole (MNI coordinates of peak voxel -52 × -12 × -37, 161 voxels) and in the fusiform gyrus (FG) extending from 40 mm from the temporal pole to 46 mm posterior to the pole (MNI coordinates of peak voxel -38 × -21 × -32, 177 voxels in cluster). Resections in the ITG were associated with an extent of BNT decline of 10.8 to 14.4% (mean 12.1%), while resection in the FG was associated with a BNT decline of 12.1 to 18.4% (mean 15.8%) (Fig. 2C). BNT decline in FG was significantly higher than in the ITG (p <0.001).

Of the 13 patients with early epilepsy manifestations, 60 – 80% received a resection within the significant regions of the ITG and FG, two patients suffered from a postoperative naming decline: one after selective transcortical amygdalohippocampectomy (with a dorsal extent from the temporal tip of 53 mm) and one after anterior temporal lobectomy (with a dorsal extent from the temporal tip of 68 mm) (Fig. 1, Supplementary material).

## 4 Discussion

### 4.1 Main findings of our study

This voxel-based lesion-symptom mapping study investigated the association of epilepsy surgery and postoperative decline in picture naming. Our findings showed that in patients with epilepsy onset after 5 years of age, resection of posterior parts of the dominant inferior temporal (ITG) and fusiform gyrus (FG) were likely to lead to postoperative decline in picture naming. The risk of deterioration in naming performance was more pronounced in the FG than in the ITG, and the extent of naming decline was greater with more temporo-posterior resections. The risk of postoperative decline of naming increased by 5% with each year of later initial manifestation.

Our findings confirm previous data that postoperative picture naming decline is related to left temporal lobe surgery (Helmstaedter, 2013, Sherman et al., 2011). In addition, our results complement previous data with a precise localization of an temporo-basal area extending from 34 to 46 mm (standardized stereotaxic space) from the temporal pole where resection is at risk of naming decline resulting in 10 – 20% postoperative naming decline across this area.

### 4.2 Localization of picture naming deficit

Neuropsychological deficit-lesion mapping showed a significant association of naming decline and resection of the ventral temporal neocortex in the area of the ITG and FG, an area corresponding to the basal temporal language area (BTLA) localized in the dominant hemisphere. Delineated by electrical cortical stimulation, this language area has an extension of 10 to 90 mm posterior to the temporal pole, comprising the ITG, FG, and parahippocampal gyrus (Krauss et al., 1996, Abdallah et al., 2021). Electrical cortical stimulation in the middle FG only disrupted naming in isolation, while other tasks, like the ability to repeat sentences, were preserved (Forseth et al., 2018). Studies using fMRI depict the dominant temporo-basal area as an essential lexical semantic hub within the language network with connections to the left anterior temporal lobe, left frontal and prefrontal regions, left angular gyrus, and occipital cortex (Binder et al., 2009, Forseth et al., 2018, Chen et al., 2019, Trimmel et al.). A strong functional connectivity to other brain regions was associated with better clinical naming performance (Trimmel et al., 2018, Trimmel et al., 2021, Trimmel et al., 2019). On the other side, damage of the white matter system could cause a disconnection of the regions involved and has shown to contribute to naming impairments as well (Kaestner et al., 2022, McDonald et al., 2008, Duffau et al., 2008). Using VBLSM, lesions affecting brain regions crucial for specific cognitive functions can be identified (Rorden et al., 2007). A correlation of naming decline with BTLA resection has been reported for both a distance of on average 25 mm and a distance of 40 to 60 mm posterior of the temporal tip (Abdallah et al., 2021, Binder et al., 2020). The critical area for picture naming of the present VBLSM study extended from 34 to 46 mm from the pole and were, thus, within the resection limits of a standard temporal resection (Wiebe et al., 2001).

### 4.3 Role of age at epilepsy onset for postoperative naming deficit

Our study also demonstrates the importance of age at epilepsy manifestation for postoperative cognitive deficits: age at seizure onset was an independent predictor of postoperative naming decline. In line with these results, age at seizure manifestation modulated our results in lesion mapping. A significant association between naming decline and lesion location was only found in the subgroup of patients with epilepsy onset after the age of 5 years (n = 108). It has been long argued that epileptic activity at a young age has the potential to induce reorganization of language (Janszky et al., 2003). However, at around 5 years of age, the development of the language network is largely at a mature stage (Springer et al., 1999, Derakhshan, 2008). Our subgroup with epilepsy onset < 5 years was significantly more likely to have a naming performance that was already abnormal preoperatively compared to patients with an onset ≥ 5 years. Early occurrence of initial precipitating injuries or epileptic activity may interrupt normal language development, which then is reflected by preoperative language deficits. This alteration may lead to weaker functional connectivity in patients with earlier epilepsy onset (Trimmel et al., 2018). In our cohort, these patients with early onset could tolerate larger temporal resections without additional postoperative naming decline. Of note, language reorganization concerns especially patients with seizure onset in early childhood (before the age of 5 or 6 years) (Krauss et al., 1996, Springer et al., 1999, Berl et al., 2014). Language dominance and typical to atypical representation is a continuum, and the organization of the language network may vary at regional levels. In addition to inter-hemispheric reorganization, there is also intra-hemispheric reorganization, for example, from temporal to extratemporal areas (Berl et al., 2014). Patients with early onset of left temporal epilepsy can present language related activation patterns with bilateral activation of ITG and increased activation of frontal language areas (Cousin et al., 2008). However, temporal regions in particular may also be affected by reorganization in epilepsy patients (Trimmel et al., 2021). Although our findings were based on a rather small cohort of 13 patients and certainly need further validation and replication in larger samples. They are in line with the assumption that in patients with seizure onset in early childhood language network is often reorganized, thus leading to atypical localization of the naming function in the left temporal areas. Precedent lesion-mapping studies provided no data on the actual range on seizure onset, leaving unclear whether patients with seizure onset before age of 5 years were included at all. Our results emphasize the age of epilepsy onset as an important factor when planning resection zones in epilepsy surgery. In patients with temporal lobe epilepsy of the dominant hemisphere with onset in early childhood, an atypical distribution of language function in the temporal lobe may be likely. Therefore, these patients might be on the safer side with regard to postoperative naming disorders after temporo-basal resections. However, in patients with epilepsy onset in later childhood, presurgical analysis of language organization on a regional level could optimize the prediction of naming deficits.

### 4.4 Risk for postoperative picture naming decline

Picture naming deteriorated in 33% of our patients 6 months after left temporal resection, which was at the lower end of a range of the risk for picture naming decline that varied from 21 to 51% in precedent studies (Helmstaedter, 2013, Sherman et al., 2011, Busch et al., 2016, Binder et al., 2020). Recent research has shown that the presence and severity of postoperative naming deficits depends on the time of testing. While early naming evaluation within one year after resections including the BTLA resulted in a naming impairment for the majority of patients, the long-term outcome observed after one year after resection of BTLA showed a partial recovery or even no lasting deficit (Abdallah et al., 2021). The time course of postoperative naming deficit is also reflected in a higher proportion of patients with deficits at an examination time point approximately 6 months postoperatively from 41 to 51% reported by Busch et al. and Binder et al. compared with a lower proportion of patients with a deficit one year postoperatively from 21 to 34% described by Helmstaedter et al. and Sherman et al. (Busch et al., 2016, Binder et al., 2020, Helmstaedter, 2013, Sherman et al., 2011).

### 4.5 Extent of postoperative picture naming decline

The BTLA does not appear to harbor exclusively picture naming functions but rather be partially compensable leading to an extent of picture naming decline of 10 to 20% but no complete anomia after resection. Because of the dynamic of neuropsychological changes following surgery, results of long-term postoperative neuropsychological assessment are more relevant, yet early postoperative deficits with the risk of partial persistence can be of relevance for the individual patient and should thus be noted (Baxendale et al., 2019, Wilson et al., 2015, Rössler et al., 2015, Trimmel et al., 2019).

### 4.6 Strengths and limitations of our study

Our study had strengths and limitations. We included a large patient cohort with a higher patient number than previous studies (Binder et al., 2020) and our cohort comprised different surgical procedures with variability in lesion location and a wide spread coverage of resections across the left temporal lobe. This may have provided a sufficient basis to detect associations between lesions and behavior. VBLSM allowed for a precise delineation of the anatomical region associated with deterioration of visual naming, showing the true functional impact of neurosurgery. There were also limitations to our work due to its monocentric, retrospective design. Naming outcome was only assessed at 6 months after surgery. Thus, we did not investigate long-term dynamics of postoperative deficits. Manual lesion delineation is observer dependent and time consuming. We explored a potential advantage of using 1x1x1mm voxels in terms of accuracy, however in comparison to a reduced resolution of 2x2x2 mm, the improvements were marginal while considerably increasing the needed time for manual tracing. A WADA test or fMRI for a reliable determination of language dominance was only performed in cases of uncertainty and not in all patients. Therefore, it cannot be excluded that a small number of patients with atypical representation were included after all. Statistical analyses were only performed in regions with a minimum of five lesions overlapped and eloquent areas tended to be avoided for clinical reasons. Although VBLSM is not only restricted to gray matter, no conclusions can be made regarding phenomena of postoperative disconnection.

## 5 Conclusions

Our study showed that postoperative naming decline can occur after epilepsy surgery in the posterior dominant ITG and FG in patients when language organization is largely complete at epilepsy onset. Resection in the dominant temporal lobe in patients with epilepsy onset after 5 years of age was associated with a higher risk of deterioration in naming performance, averaging from 10 to 20%, and was more pronounced in the FG as compared to the ITG.

## Declaration of Competing Interest

The authors declare the following financial interests/personal relationships which may be considered as potential competing interests: HMH has served on the scientific advisory board of Angelini, Bittium, Corlieve, Eisai, GW, Sandoz, UCB Pharma and Zogenix. He served on the speakers’ bureau of or received unrestricted grants from Ad-Tech, Alnylam, Bracco, Desitin, Eisai, GW, Micromed, Nihon Kohden, Novartis, Pfizer, and UCB Pharma. JDL served on the speakers’ bureau of Eisai and Desitin and received a travel grant from Eisei. FS received speaker honoraria from Teva, Lilly and Novartis. RT received speaker honoraria from PCT Pharma, Desitin and Eisei.

## Figures and Tables

**Supplementary figure 1:**
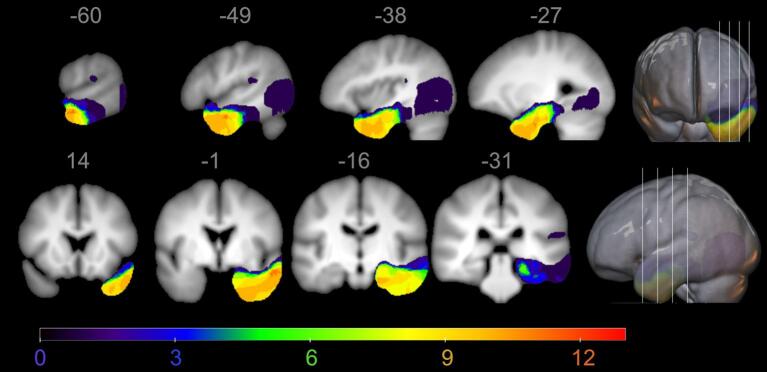


## References

[b0005] Wiebe S., Blume W.T., Girvin J.P., Eliasziw M. (2001). A randomized, controlled trial of surgery for temporal-lobe epilepsy. N. Engl. J. Med. [Internet]..

[b0010] Josephson C.B., Dykeman J., Fiest K.M., Liu X., Sadler R.M., Jette N. (2013). Systematic review and meta-analysis of standard vs selective temporal lobe epilepsy surgery. Neurology..

[b0015] Drane D.L., Loring D.W., Voets N.L., Price M., Ojemann J.G., Willie J.T. (2015). Better object recognition and naming outcome with MRI-guided stereotactic laser amygdalohippocampotomy for temporal lobe epilepsy. Epilepsia..

[b0020] Ross L., Naduvil A.M., Bulacio J.C., Najm I.M., Gonzalez-Martinez J.A. (2018). Stereoelectroencephalography-guided laser ablations in patients with neocortical pharmacoresistant focal epilepsy: concept and operative technique. Oper Neurosurg..

[b0025] Liu D.D., Lauro P.M., Phillips R.K., Leary O.P., Zheng B., Roth J.L. (2021). Two-trajectory laser amygdalohippocampotomy: Anatomic modeling and initial seizure outcomes. Epilepsia..

[b0030] Helmstaedter C. (2013). Cognitive outcomes of different surgical approaches in temporal lobe epilepsy. Epileptic Disord..

[b0035] Baxendale S., Thompson P. (2018). Red flags in epilepsy surgery: Identifying the patients who pay a high cognitive price for an unsuccessful surgical outcome. Epilepsy Behav. [Internet]..

[b0040] Khoo A., de Tisi J., Mannan S., O’Keeffe A.G., Sander J.W., Duncan J.S. (2021). Reasons for not having epilepsy surgery. Epilepsia..

[b0045] Krauss G.L., Fisher R., Plate C., Hart J., Uematsu S., Gordon B. (1996). Cognitive effects of resecting basal temporal language areas. Epilepsia..

[b0050] Sherman E.M.S., Wiebe S., Fay-Mcclymont T.B., Tellez-Zenteno J., Metcalfe A., Hernandez-Ronquillo L. (2011). Neuropsychological outcomes after epilepsy surgery: Systematic review and pooled estimates. Epilepsia..

[b0055] Lüders H., Lesser R.P., Hahn J., Dinner D.S., Morris H.H., Wyllie E. (1991). Basal temporal language area. Brain..

[b0060] Bédos Ulvin L., Jonas J., Brissart H., Colnat-Coulbois S., Thiriaux A., Vignal J.P. (2017). Intracerebral stimulation of left and right ventral temporal cortex during object naming. Brain Lang [Internet]..

[b0065] Abdallah C., Brissart H., Colnat-Coulbois S., Pierson L., Aron O., Forthoffer N. (2021). Stereoelectroencephalographic language mapping of the basal temporal cortex predicts postoperative naming outcome. J. Neurosurg..

[b0070] Binder J.R., Desai R.H., Graves W.W., Conant L.L. (2009). Where is the semantic system? A critical review and meta-analysis of 120 functional neuroimaging studies. Cereb. Cortex.

[b0075] Forseth K.J., Kadipasaoglu C.M., Conner C.R., Hickok G., Knight R.T., Tandon N. (2018). A lexical semantic hub for heteromodal naming in middle fusiform gyrus. Brain..

[b0080] L. Chen D. Wassermann D.A. Abrams J. Kochalka G. Gallardo-Diez V. Menon The visual word form area (VWFA) is part of both language and attention circuitry Nat. Commun. [Internet]. 10 1 2019 1 12. Available from: http://dx.doi.org/10.1038/s41467-019-13634-z. 10.1038/s41467-019-13634-z PMC6898452 31811149

[b0085] Helmstaedter, C., Witt, J.A. Clinical neuropsychology in epilepsy: Theoretical and practical issues [Internet]. 1st ed. Vol. 107, Handbook of Clinical Neurology. Elsevier B.V.; 2012. 437–459 p. Available from: http://dx.doi.org/10.1016/B978-0-444-52898-8.00036-7. 10.1016/B978-0-444-52898-8.00036-7 22938988

[b0090] Busch R.M., Floden D.P., Prayson B., Chapin J.S., Kim K.H., Ferguson L. (2016). Estimating risk of word-finding problems in adults undergoing epilepsy surgery. Neurology..

[b0095] Rorden, C., Karnath, H., Bonilha, L. Improving Lesion – Symptom Mapping. 2007: 1081–8. 10.1162/jocn.2007.19.7.1081 17583985

[b0100] Binder J.R., Tong J.Q., Pillay S.B., Conant L.L., Humphries C.J., Raghavan M. (2020). Temporal lobe regions essential for preserved picture naming after left temporal epilepsy surgery. Epilepsia..

[b0105] Kaplan, E., Goodglass, H., Weintraub, S., & Goodglass, H. Boston naming test. 183AD; Philadelph.

[b0110] Engel, J. Update on surgical treatment of the epilepsies. Summary of the Second International Palm Desert Conference on the Surgical Treatment of the Epilepsies (1992). Neurology [Internet]. 1993 [cited 2021]; 43(8):1612–7. Available from: https://pubmed.ncbi.nlm.nih.gov/8102482/. 10.1212/wnl.43.8.1612 8102482

[b0115] Baxendale S., Wilson S.J., Baker G.A., Barr W., Helmstaedter C., Hermann B.P. (2019). Indications and expectations for neuropsychological assessment in epilepsy surgery in children and adults: Executive summary of the report of the ILAE Neuropsychology Task Force Diagnostic Methods Commission: 2017–2021. Epilepsia..

[b0120] Nicholas L.E., Brookshire R.H., MacLennan D.L., Schumacher J.G., Porrazzo S.A. (1988). The Boston Naming Test: Revised Administration and Scoring Procedures and Normative Information for Non-Brain-Damaged Adults. Clinical. Aphasiology..

[b0125] Sawrie SM, Chelune GJ, Naugle RI, Lüders HO. Empirical methods for assessing meaningful neuropsychological change following epilepsy surgery. J Int Neuropsychol Soc [Internet]. 1996 [cited 2022]; 2(6):556–64. Available from: https://www.cambridge.org/core/journals/journal-of-the-international-neuropsychological-society/article/abs/empirical-methods-for-assessing-meaningful-neuropsychological-change-following-epilepsy-surgery/0A4CB8B77B29E733B0D187B24A4AC8A7. 10.1017/s1355617700001739 9375160

[b0130] Zentner J. (2016). Chirurgische Epilepsietherapie. Zeitschrift für Epileptol.

[b0135] Rorden C., Bonilha L., Fridriksson J., Bender B., Karnath H.O. (2012). Age-specific CT and MRI templates for spatial normalization. Neuroimage..

[b0140] Karnath H.O., Sperber C., Wiesen D., de Haan B. (2020). Lesion-behavior mapping in cognitive neuroscience: A practical guide to univariate and multivariate approaches. Neuromethods..

[b0145] Rorden C, Brett M. Stereotaxic display of brain lesions. [cited 2022]; . Available from: www.fil.ion.ucl.ac.uk/spm/. 10.1155/2000/421719 11568431

[b0150] Nachev P., Coulthard E., Jäger H.R., Kennard C., Husain M. (2008). Enantiomorphic normalization of focally lesioned brains. Neuroimage..

[b0155] Ashburner J., Friston K.J. (2005). Unified segmentation. Neuroimage..

[b0160] von Elm E., Altman D.G., Egger M., Pocock S.J., Gøtzsche P.C., Vandenbroucke J.P. (2008). The Strengthening the Reporting of Observational Studies in Epidemiology (STROBE) statement: guidelines for reporting observational studies. J. Clin. Epidemiol..

[b0165] Berl M.M., Zimmaro L.A., Khan O.I., Dustin I., Ritzl E., Duke E.S. (2014). Characterization of atypical language activation patterns in focal epilepsy. Ann. Neurol..

[b0170] Trimmel, K., Vos, S.B., Caciagli, L., Xiao, F., Graan, L.A., Van Winston, G.P., et al. Decoupling of functional and structural language networks in temporal lobe epilepsy. 10.1111/epi.17098 PMC8776336 34642939

[b0175] Trimmel K., Van Graan A.L., Caciagli L., Haag A., Koepp M.J., Thompson P.J. (2018). Left temporal lobe language network connectivity in temporal lobe epilepsy. Brain..

[b0180] Trimmel, K., Caciagli, L., Xiao, F., van Graan, L.A., Koepp, M.J., Thompson, P.J., et al. Impaired naming performance in temporal lobe epilepsy: language fMRI responses are modulated by disease characteristics. J Neurol [Internet]. 2021; 268(1):147–60. Available from: https://doi.org/10.1007/s00415-020-10116-x. 10.1007/s00415-020-10116-x PMC7815622 32747979

[b0185] Trimmel K., van Graan L.A., Gonzálvez G.G., Haag A., Caciagli L., Vos S.B. (2019). Naming fMRI predicts the effect of temporal lobe resection on language decline. Ann. Clin. Transl. Neurol..

[b0190] Kaestner, E., Stasenko, A., Ben-Haim, S., Shih, J., Paul, B.M., McDonald, C.R. The importance of basal-temporal white matter to pre- and post-surgical naming ability in temporal lobe epilepsy. NeuroImage Clin [Internet]. 2022; 34(September 2021):102963. Available from: https://doi.org/10.1016/j.nicl.2022.102963. 10.1016/j.nicl.2022.102963 PMC8888987 35220106

[b0195] McDonald C.R., Ahmadi M.E., Hagler D.J., Tecoma E.S., Iragui V.J., Gharapetian L. (2008). Diffusion tensor imaging correlates of memory and language impairments in temporal lobe epilepsy. Neurology..

[b0200] Duffau H., Thiebaut De Schotten M., Mandonnet E. (2008). White matter functional connectivity as an additional landmark for dominant temporal lobectomy. J. Neurol. Neurosurg. Psychiatry.

[b0205] Janszky J., Jokeit H., Heinemann D., Schulz R., Woermann F.G., Ebner A. (2003). Epileptic activity influences the speech organization in medial temporal lobe epilepsy. Brain..

[b0210] Springer J.A., Binder J.R., Hammeke T.A., Swanson S.J., Frost J.A., Bellgowan P.S.F. (1999). Language dominance in neurologically normal and epilepsy subjects. A functional MRI study. Brain..

[b0215] Derakhshan I. (2008). Atypical language in lesional and nonlesional complex partial epilepsy. Neurology..

[b0220] Cousin, E., Baciu, M., Pichat, C., Kahane, P., Le Bas, J.F. Functional MRI evidence for language plasticity in adult epileptic patients: Preliminary results. Neuropsychiatr Dis Treat. 2008; 4(1 B):235–46. 10.2147/ndt.s2330 PMC2515912 18728818

[b0225] Wilson S.M., Lam D., Babiak M.C., Perry D.W., Shih T., Hess C.P. (2015). Transient aphasias after left hemisphere resective surgery. J. Neurosurg..

[b0230] Rössler K., Sommer B., Grummich P., Hamer H.M., Pauli E., Coras R. (2015). Risk reduction in dominant temporal lobe epilepsy surgery combining fMRI/DTI Maps, neuronavigation and intraoperative 1.5-tesla MRI. Stereotact. Funct. Neurosurg..

