# Supplementary material for: Resection of dominant fusiform gyrus is associated with decline of naming function when temporal lobe epilepsy manifests after the age of five: A voxel-based lesion-symptom mapping study
Source: Neuroimage Clin. 2022 Jul 29;35:103129. doi: 10.1016/j.nicl.2022.103129 (PMC9421498; doi:10.1016/j.nicl.2022.103129)

**SUPPORTING INFORMATION**

**Table 1: Neuropsychological data of patients with left temporal epilepsy surgery- BNT preoperative, postoperative and change score**

|  |  | Pre | |  | Post | |  | % Change | |
| --- | --- | --- | --- | --- | --- | --- | --- | --- | --- |
| Resections type | N | M (SD) | impaired  n (%) |  | M (SD) | impaired  n (%) |  | M (SD) | impaired  n (%) |
| Hippocampal |  |  |  |  |  |  |  |  |  |
| ATL | 26 | 43.7 (11.3) | 13 (50) |  | 37.2 (11.8) | 23 (89) |  | -14.7 (17.9) | 15 (58) |
| Pole + AH | 29 | 44.6 (7.7) | 17 (59) |  | 42.4 (7.8) | 21 (72) |  | -4.0 (15.1) | 9 (31) |
| Selective AH | 10 | 46.3 (8.9) | 6 (60) |  | 43.4 (8.0) | 7 (70) |  | -4.5 (17.1) | 3 (30) |
| Neocortical | 56 | 49.1 (7.8) | 13 (23) |  | 47.1 (7.5) | 23 (41) |  | -3.2 (11.6) | 13 (23) |
| Total | 121 | 46.6 (8.9) | 59 (49) |  | 43.5 (9.4) | 80 (66) |  | -6.0 (15.0) | 40 (33) |

Abbreviations: ATL anterior temporal lobe, AH amygdalohippocampal, n number, M median, SD standard deviation

**Table 2: Number of Wada-test/fMRI stratified by handedness and location of the resection**

|  |  |  | Handedness |  |  |  |  |
| --- | --- | --- | --- | --- | --- | --- | --- |
|  | right |  | ambidextrous |  | left |  | total |
| Resection | n Wada/fMRI per subgroup (%) |  | n Wada/fMRI per subgroup (%) |  | n Wada/fMRI per subgroup (%) |  | n Wada/fMRI per subgroup (%) |
| Left temporal | 53/106  (50%) |  | 2/5  (40%) |  | 9/10  (90%) |  | 64/121  (53%) |
| Left extratemporal | 3/17  (18%) |  | 1/1  (100%) |  | 1/2  (50%) |  | 5/20  (25%) |
| Right temporal | 24/127  (19%) |  | 2/3  (67%) |  | 6/10  (60%) |  | 32/140  (23%) |
| Right extratemporal | 2/28  (7%) |  | - |  | 2/2  (100%) |  | 4/30  (13%) |
| Total | 82/278  (29%) |  | 5/9  (56%) |  | 18/24  (75%) |  | 105/311  (34%) |

Figure 1: Lesion overlap map of patients with left temporal resection and epilepsy onset before the age of 5 (n=13), that were excluded from VBLSM of patients with left temporal resections. Color bar visualizes number of patients with overlapping resection zones. Coordinates are presented in MNI space.


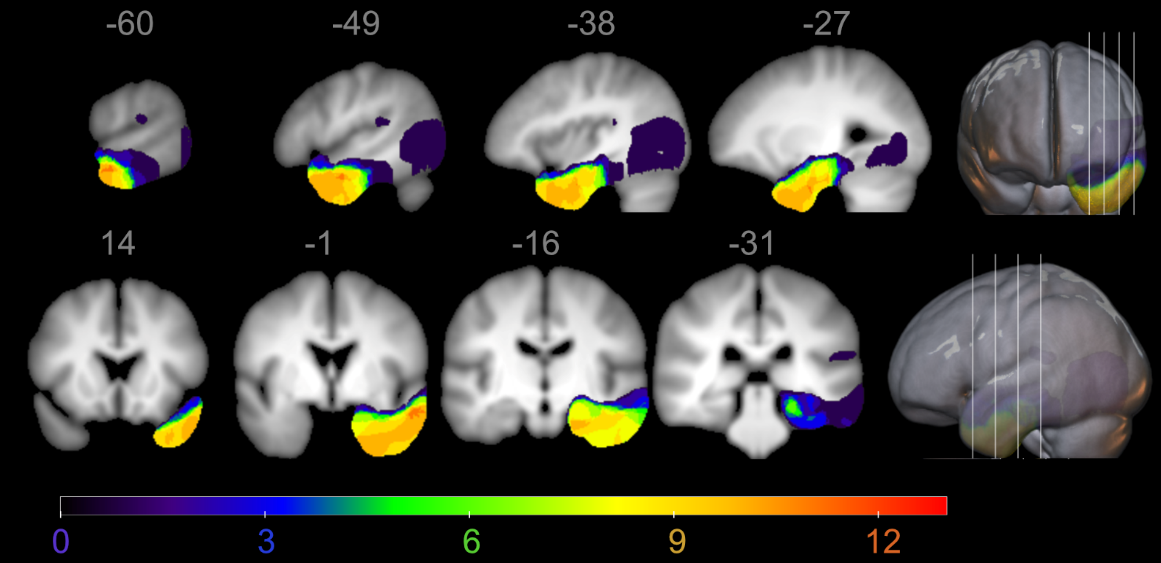

Supplement: Supplementary data 1 [file mmc1.docx]
